# Patterns of informal family care during acute hospitalization of older adults from different ethno-cultural groups in Israel

**DOI:** 10.1186/s12939-020-01314-0

**Published:** 2020-11-23

**Authors:** Ksenya Shulyaev, Nurit Gur-Yaish, Efrat Shadmi, Anna Zisberg

**Affiliations:** 1 The Cheryl Spencer Department of Nursing, Faculty of Social Welfare and Health Science, Haifa University, Mt. Carmel, 3498838 Haifa, Israel; 2 The Center for Research and Study of Aging, Faculty of Social Welfare and Health Science, Haifa University, Haifa, Israel; 3 Oranim Academic College of Education, Kiryat Tiv’on, Israel

## Abstract

**Introduction:**

Informal caregiving during hospitalization of older adults is significantly related to hospital processes and patient outcomes. Studies in home settings demonstrate that ethno-cultural background is related to various aspects of informal caregiving; however, this association in the hospital setting is insufficiently researched.

**Objectives:**

Our study explore potential differences between ethno-cultural groups in the amount and kind of informal support they provide for older adults during hospitalization.

**Methods:**

This research is a secondary data analysis of two cohort studies conducted in Israeli hospitals. Hospitalized older adults are divided into three groups: Israeli-born and veteran immigrant Jews, Arabs, and Jewish immigrants from the Former Soviet Union (FSU). Duration of caregiver visit, presence in hospital during night hours, type of support (using the Informal Caregiving for Hospitalized Older Adults scale) are assessed during hospitalization. Results are controlled by background parameters including functional Modified Barthel Index (MBI) and cognitive Short Portable Mental Status Questionnaire (SPMSQ) status, chronic morbidity (Charlson), and demographic characteristics.

**Results:**

Informal caregivers of “FSU immigrants” stay fewer hours during the day in both cohorts, and provide less supervision of medical care in Study 2, than caregivers in the two other groups. Findings from Study 1 also suggest that informal caregivers of “Arab” older adults are more likely to stay during the night than caregivers in the two other groups**.**

**Conclusions:**

Ethno-cultural groups differ in their patterns of caregiving of older adults during hospitalization. Health care professionals should be aware of these patterns and the cultural norms that are related to caregiving practices for better cooperation between informal and formal caregivers of older adults.

## Introduction

### Background

When older adults are hospitalized, family members and friends commonly provide care and support. International data show that 77 to 96% of older adults are accompanied by family caregivers during a hospital stay and that these caregivers stay in the hospital for an average of 5 h per day [1–3]. They provide a variety of support: instrumental support (help with eating, bathing, and other activities of daily living), supervision of instrumental support given by hospital staff, ensuring/supervision of medical care, and psychological support [4]. These types of support have been related to patients’ clinical and personal characteristics [5]. For example, patients with lower functioning levels require more instrumental care, whereas those with anxiety require more psychological support [4].

Results of recent studies show that family caregiving is related to both the in-hospital processes of care and the health outcomes of older adults. Patterns of family caregiving during hospitalization are associated with eating and walking during hospitalization [6], treatment adherence [7, 8], level of depressive symptoms [9], functional decline [10], length of stay in hospital, re-hospitalization, and mortality [11, 12]. Therefore, understanding the patterns and characteristics of family caregiving may help optimize the processes of care and improve outcomes of hospitalization for older adults.

Patients’ and families’ ethno-cultural backgrounds are known to play a role outside the hospital. Ethno-cultural groups differ in their norms, values, and attitudes with respect to supporting older adults [13–17]. Studies in home-care settings suggest that these differences are manifested in the scope and diversity of family caregivers’ networks, the amount of time they provide care, and their resources and levels of psychological burden [18–20]. However, the association between ethno-cultural background and various aspects of informal care may differ in the hospital setting. First, hospitalization brings together two cultural contexts of care: the personal and the organizational. These coexisting contexts might pose challenges for patients and caregivers from minority groups [21, 22]. Second, the stressful aspects of hospitalization might require different coping strategies from different ethno-cultural groups. For example, studies show that an avoidant coping strategy is salient in caregivers in some groups (i.e., African Americans) but not others (i.e., Korean-origin caregivers) [23]. This suggests the need to investigate the degree to which ethno-cultural groups differ in their provision of family caregiving in the unique context of the hospital.

Israeli society includes a variety of national, religious, secular, and ethnically unique population groups and, as such, provides an ideal setting for this type of study. We focused on Arabs, Jewish immigrants from the Former Soviet Union (FSU), and Israeli-born or veteran immigrant Jews, defining veterans as those arriving before 1989, when major flows from the FSU began. Arabs and FSU immigrants comprise the largest minority groups in the Israeli older adult population (13 and 18.8%, respectively) [24]. The decision to study these three groups was based on the cultural diversity and distinctiveness of their family cultures [25], attitudes toward older adults [26] intergenerational relationships [27, 28], expectations and patterns of in-home support [29], and utilization of health care services [30, 31]. These differences have been established in home-care settings, but to the best of our knowledge, only one study has examined ethno-cultural aspects of caregiving during hospitalization. This study suggested that patients from the FSU were less likely to have informal caregivers visit them during the hospital stay than were Israeli-born or veteran immigrant Jews and Arabs [32].

#### Objectives

Our study explored potential differences between these three groups in the amount and kind of informal support they provided older adults during hospitalization. Specifically, we hypothesized that there would be differences in the number of hours that family caregivers visited patients during the day and in whether family caregivers stayed with patients during the night. We also hypothesized differences in the kind of support provided, that is, in instrumental support, supervision of instrumental support, ensuring/supervision of medical care, and psychological support, even after controlling for patients’ clinical and personal characteristics. Possible findings could be useful for raising awareness among health care workers of the characteristics of ethno-cultural groups and could help provide culture-appropriate care and communication with family caregivers.

## Methods

### Study design and settings

This study was a secondary data analysis of two cohort studies conducted in internal medicine units in hospitals in northern Israel. We used unrelated cohorts from two different studies with a similar methodology, but in different hospitals and different populations, to identify persistent cultural patterns in informal caregiving during hospitalization. The first study was the “HoPE-FOR” (Hospitalization Process Effects on Functional Outcomes and Recovery) study conducted in two hospitals in a large city (around 280,000 citizens) from February 2009 to August 2011 [33]. The second was the “Walk-FOR” (Functional Outcomes and Recovery) intervention to increase mobility study (including intervention and control groups), conducted in one hospital in a peripheral city (around 40,000 citizens) from October 2015 to September 2016 [34]. These hospitals serve diverse population groups, and each has unique characteristics related to the urban/rural communities surrounding it.

#### Participants

The two studies’ populations included older adults urgently admitted for nondisabling diagnoses who were able to communicate in Hebrew, Russian, or Arabic and to walk 2 weeks before hospitalization. Only cognitively competent patients (scoring 5 or above on Pfeiffer’s Short Portable Mental Status Questionnaire (SPMSQ) [33] participated.

#### Validation and ethics

All questionnaires were written in Hebrew and then translated into Russian and Arabic. Process linguistic validation (to ensure that the same intended meaning of each element of each questionnaire remained intact), cross-cultural adaptation of Russian and Arabic versions, and cross-cultural validation of research instruments translation included five stages: (1) two Hebrew-Russian and two Hebrew-Arabic speakers independently translated questionnaires, (2) and compared the two translated versions; (3) two other translators conducted blind back-translations for each language; after which (4) items showing change after translation were discussed until an equivalent meaning was reached in both languages; and (5) revised items were translated into Hebrew and again compared by a group of bilingual researchers [36]. The studies were approved by the ethics committees of the hospitals and by the university review board. All participants provided informed consent, and participation was voluntary and confidential.

Items showing change after translation were discussed until an equivalent meaning was reached in both languages. Revised items were translated into Hebrew and again compared.

#### Statistical analyses

Means and standard deviations (SDs) were computed for all continuous study variables. The associations between the independent variable (belonging to the ethno-cultural group), dependent variables (duration and kind of informal support), and control variables were examined using multivariate analysis of covariance (MANCOVA) and logistic regression analyses. Because of the multiple tests, we ran a Bonferroni correction for the significance of the models. All *p*-values remained below .05. In all analyses, we controlled for the following: age, gender, family status, and number of children; cognitive, functional, and psychological (anxiety and depression) status; economic status; length of stay; severity of illness and comorbidity that founded to be related to amount of support [4]. All analyses were conducted using IBM SPSS Statistics version 24.

## Study 1

### Methods

#### Participants

Of the 1042 older patients who met the inclusion criteria of the initial studies, 241 had no information on family caregiving, and 139 were excluded because of low cognitive status, resulting in a final sample of 662 participants. The functional status before hospitalization, number of children, education, perceived economic status, family status, level of depression and anxiety, gender, and length of hospital stay of the excluded participants did not differ significantly from those of participants retained in the final sample. However, the excluded individuals were older (*p* < .05), had lower cognitive status (*p* < .05), and a higher level of comorbidity (*p* < .05).

#### Measures

Informal support was assessed with the Informal Caregiving for Hospitalized Older Adults (ICHOA) scale [4]. This measure consists of four subscales: instrumental support (e.g., help with eating or grooming), supervision of instrumental support (e.g., making sure staff help with eating), ensuring/supervision of medical care (e.g., monitoring medical care or discussing patient’s condition with medical staff), and psychological support (e.g., comforting patients when sad or helping them get used to the hospital). All subscales include 3 to 6 items evaluated on a Likert-type response scale from 1 (*did not receive any help*) to 5 (*received help all the time*). We calculated the mean for each subscale, and this continuous variable was used in further analysis as a level of this kind of support. For this study, the reliability of scale items was good (from α = .83 to α = .88). Length of family caregivers’ visits during the day was self-reported by patients and measured in hours. Family caregiver stay during the night was dichotomized as 0 (*did not stay with the patient during the night*) and 1 (*accompanied during the night for at least 4 hours*)..

#### Ethno-cultural groups

Participants were divided into three ethno-cultural groups according to their self-identified native language (Hebrew, Arabic, or Russian), religion (Jews, Christian, or Muslim), country of birth (Israel, USSR, or other countries), and year of immigration (before or after 1989, with the former considered veteran immigrants). The three groups were as follows: (1) “Jews”, Israeli-born with Hebrew as a mother language and/or veteran immigrants arriving before 1989; (2) “Arabs”, Israeli-born with Arabic as a mother language and Christian or Muslim religion; and (3) “FSU immigrants”, immigrants to Israel from FSU with Russian as their native language.

#### Background characteristics

Sociodemographic variables and variables related to functional, cognitive, psychological, and health status were included in the data analysis as potential confounders. *Functional status* on admission was assessed using the 11-item Modified Barthel Index (MBI) [37], consisting of individuals’ self-assessment of their independence in performing basic activities of daily living. *Cognitive status* was measured using the Pfeiffer Short Portable Mental Status Questionnaire (SPMSQ) [35]. In this questionnaire, total scores range from 0 to 10; higher scores indicate better cognitive status. *Level of anxiety* was assessed using the Short Anxiety Screening Test (SAST) [38], a 10-item, 4-point Likert-scale questionnaire; anxiety is defined as scoring 24 points or higher. *Level of depression* was measured using the 10-item Short Zung Interviewer Assisted Depression Rating Scale (Short Zung IDS) [39]. Responses are rated on a 4-point scale, with the total score recoded from 0 to 75; depression is defined as scoring 70 points or more on the scale. Severity of acute health conditions was measured with the Acute Physiology and Chronic Health Evaluation (APACHE II) [40], a valid and reliable assessment of severity of illness in acute conditions. The APACHE II uses a point score (range 0–71) based on the initial values of 12 routine physiological measurements, age, and previous health status to provide a general measure of severity of disease. *Severity of chronic health condition* was assessed using Charlson’s Comorbidity Index; the index weights 20 health conditions and their severity on a scale from 1 to 6 [41]. The sociodemographic variables age, gender, family status, number of children, and economic status were also included. Economic status was estimated by self-report on a scale from 1 (*worst*) to 5 (*best*).

#### Procedure

Functional and cognitive status, as well as background characteristics, were assessed within the first 24 h of hospital admission. In-hospital informal support was assessed via in-person interviews at the end of hospitalization. Data on chronic morbidity and length of stay were retrieved from the hospitals’ electronic medical records.

### Results

#### Participants’ characteristics

The mean age of participants was 78.1 (*SD* = 5.7), half were male (51.4%, *N* = 340), and half were married (54.7%, *N* = 362). The mean number of children was 2.8 (*SD* = 2.1). Average cognitive and functional statuses were relatively high, indicating a mostly independent sample (cognitive status, *M* = 8.7, *SD* = 1.5; functional status before hospitalization, *M* = 91.7, *SD* = 15.4). About one-fifth screened positively for anxiety (16.8%, *N* = 110) and depression (20%, *N* = 131). Subjective economic status of most participants was “like others” (42.1%, *N* = 270) or higher (39.3%, *N* = 252). Average length of hospital stay was 6.3 days (*SD* = 5.2). Average comorbidity score was 2.5 (*SD* = 2.1) (see Table 1).

**Table 1:** Study 1: Participants’ characteristics (*N* = 662)

	“Jews” (*N* = 420)	“Arabs” (*N* = 40)	“FSU immigrants” (*N* = 202)	Total
	*M* (*SD*)	*M* (*SD*)	*M* (*SD*)	*M* (*SD*)
Age	78.5 (5.7)	75.3 (4.9)	77.8 (5.7)	78.1 (5.7)
Gender: Male N(%)	225 (53.6%)	33 (82.5%)	82 (40.6%)	340 (51.4%)
Family status: Married N(%)	229 (54.5%)	34 (85%)	99 (49.0%)	362 (54.7%)
Number of children	2.9 (1.8)	6.7 (3.2)	1.8 (1.2)	2.8 (2.1)
Cognitive status: SPMSQ (0–10)	8.6 (1.4)	8.4 (1.8)	8.9 (1.5)	8.7 (1.5)
Functional status 2 weeks before hospitalization: MBI (0–100)	90.4 (17.1)	89.5 (17.2)	94.8 (9.9)	91.7 (15.4)
Anxiety: SAST (10–40)	17.2 (4.8)	17.3 (4.7)	18 (4.9)	19.1 (5.3)
Depression: Short Zung IDS (25–100)	20.9 (6.2)	22.1 (6.5)	20.6 (6)	52.3 (15.3)
Economic status (1–5)	3.3 (0.9)	3 (1.1)	3 (0.9)	3.2 (1.0)
Length of stay in hospital (days)	6.3 (5.4)	7.9 (7.8)	5.8 (3.7)	6.3 (5.2)
Severity of illness: APACHE II (0–71)	11.6 (4.4)	11.7 (3.9)	10.9 (3.9)	11.4 (4.3)
Comorbidity score: Charlson (0–22)	2.9 (1.8)	6.7 (3.2)	1.8 (1.2)	2.5 (2.1)

#### Preliminary analysis

All background characteristics (age, gender, family status, number of children, and functional, cognitive, psychological, and health status) were significantly related to at least one of the study variables and thus were included as control variables.

#### Ethno-cultural sample characteristics

Most participants were non- or veteran immigrant Jews (63.5%, *N* = 420), more than a quarter were “FSU immigrants” (30.5%, *N* = 202), and 6% (*N* = 40) were “Arabs.”

#### Informal caregiving during hospitalization

##### Hours of day visits

Almost all participants (97%, *N* = 622) were accompanied by family caregivers during their hospitalization. The average length of day visits was 4.7 h (*SD* = 3.5).

##### Night stay

Only 7.9% of participants (*N* = 52) were accompanied by family caregivers during the night (a “stay” was defined as at least 4 h).

##### Type of support

Less than half the participants (34.3%, *N* = 229) received any instrumental support from their family caregivers during hospitalization, and only 11.6% (*N* = 77) reported that their family caregivers supervised instrumental support from the hospital staff. Psychological support was the most prevalent: 94.5% (*N* = 624) of hospitalized elders reported receiving such support from family caregivers during hospitalization. The majority (83.1%, *N* = 551) reported that their family caregivers ensured/supervised medical care.

#### Ethno-cultural differences in informal caregiving

##### Hours of day visits

MANCOVA analyses controlling for all covariates revealed significant differences between the ethno-cultural groups (*F* (2, 615) = 13.86, *p* < .0005). Bonferroni post-hoc analysis showed that the “FSU immigrants” had the shortest visits (estimated average 3.6 h per day), shorter than “Jews” (5.2 h per day, *p* < .0005) and “Arabs” (5.8 h per day, *p* = .007) (Table 2).

**Table 2:** Study 1: Ethno-cultural differences in informal caregiving (*N* = 662)

	“Jews” (*N* = 420)	“Arabs” (*N* = 40)	“FSU immigrants” (*N* = 202)
	*M* (*SD*)^e^	*M* (*SD*)^e^	*M* (*SD*)^e^
Day visit (hours)	5.2 (0.2)^a^	5.8 (0.6) ^b^	3.6 (0.3)^a,b^
Instrumental support (1–5)	1.7 (0.1)	1.7 (0.2)	1.7 (0.1)
Supervision instrumental support (1–5)	1.2 (0.03)	1.2 (0.1)	1.2 (0.1)
Ensuring/supervision medical care (1–5)	3.3 (0.7)	2.8 (0.3)	3.2 (0.1)
Psychological support (1–5)	4.0 (0.1) ^c^	3.7 (0.2) ^d^	4.2 (0.1)^c,d^

^a^*p* < 0.001

^b^*p* < 0.01

^c^*p* < 0.01

^d^*p* < 0.05

^e^Adjusted for covariates: age, gender, family status, number of children; cognitive, functional, psychological (anxiety and depression), and economic status; length of stay; severity of illness and comorbidity

##### Night stay

Logistic regression analysis showed that “Arabs” were more likely to be accompanied by family caregivers during the night hours than were “Jews” (OR = 3.12; CI 95% (1.03–9.41); *p* = .044) or “FSU immigrants” (OR = 7.32; CI 95% (1.62–33.0); *p* = .01).

##### *Differences in kinds of support*

MANCOVA analyses controlling for all covariates showed that “FSU immigrants” received significantly more psychological support from family caregivers (*M* = .2, *SD* = 0.1) than did both “Jews” (*M* = 4.2, *SD* = 0.1, *p* < 0.01) and “Arabs” (*M* = 3.7, *SD* = 0.1, *p* < 0.05). There were no significant differences between the three ethno-cultural groups for other kinds of informal support (Table 2).

## Study 2

### Methods

#### Procedure

Functional and cognitive status, background characteristics, chronic morbidity, and length of stay were assessed as in Study 1, with the same instruments. In-hospital informal support was assessed using in-person daily interviews (up to three follow-up interviews during the hospital stay, *M* days of follow-ups = 2.17, *SD* = .84).

#### Participants

The participants’ recruitment and dropout process was described previously (BLINDED FOR REVIEW). Of 401 older patients who met the inclusion criteria for the initial study, 14 lacked information about family caregiving, resulting in a final sample of 387 participants. Excluded participants were comparable to participants retained in the final sample for the following: age, gender, family status, and number of children; functional and psychological (anxiety and depression) status; economic status; comorbidity. However, excluded individuals had a shorter hospital stay (*p* < .05) and lower cognitive status (*p* < .05).

#### Measures

*Informal support* was assessed with the ICHOA scale [4]. The reliability of subscales in the current study ranged from α = .84 to α = .89 in the three follow-ups. The length of family caregivers’ visits during the days and the night stay were estimated as in Study 1. The total support received by patients was computed as the mean for each subscale of up to three follow-ups.

##### Ethno-cultural groups

Participants were divided into the three ethno-cultural groups described in Study 1.

##### Background characteristics

The following variables were assessed as in Study 1: age, gender, family status, and number of children; cognitive and functional status; length of hospital stay; comorbidity. *Severity of acute health conditions* was assessed with the National Early Warning Score (NEWS) [42], a well-validated, track-and-trigger, early-warning score system that is used to identify and respond to patients at risk of deteriorating. It is based on a simple scoring system in which a score is given to physiological measurements already being monitored when patients are in health care settings. A summary score ranges from 0 (*low risk*) to 20 (*high risk*). *Psychological status* was measured using the Hospital Anxiety and Depression Scale (HADS). The HADS includes two subscales, for anxiety and depression. Each contains seven items. These were rated from 0 to 3, and the total score was dichotomized according to the cut-point [43]*. Economic status* was self-reported on the following subjective 3-point scale: “If the average income of a family in Israel is 10000 NIS, your income is higher than average (1), similar to average (2), or lower than average (3).” We also controlled for participation in the larger study’s intervention group to make sure that participating in the intervention did not affect the results in any way.

### Results

#### Participants’ characteristics

Participants’ mean age was 75.4 (*SD* = 7.1). Most participants were male (59.9%, *N* = 232), and most were married (58.4%, *N* = 226). The mean number of children was 4.8 (*SD* = 3.0). The average cognitive and functional statuses were relatively high (cognitive status *M* = 9.1, *SD* = 1.4; functional status, *M* = 89.6, *SD* = 16.0). About one-fifth suffered from anxiety (22.5%, *N* = 87), and about one-third, from depression (34.1%, *N* = 132). The self-reported economic status of most participants was “lower than average” (84.8%). Average length of hospital stay was 6.2 days (*SD* = 3.7), and average comorbidity score was 2.2 (*SD* = 1.9) (Table 3).

**Table 3:** Study 2: Participants’ characteristics (*N* = 387)

	“Jews” (*N* = 209)	“Arabs” (*N* = 127)	“FSU immigrants” (*N* = 51)	Total
	*M* (*SD*)	*M* (*SD*)	*M* (*SD*)	*M* (*SD*)
Age	78.1 (6.4)	74.7 (6.4)	78.1 (6.2)	75.4 (7.1)
Gender: Male N(%)	121 (57.9%)	86 (67.7%)	25 (49%)	232 (59.9%)
Family status: Married N(%)	119 (56.9%)	83 (65.4%)	44 (34.6%)	226 (58.4%)
Number of children	3.2 (2.1)	7.1 (3.1)	1.8 (1.1)	4.8 (3.0)
Cognitive status: SPMSQ (0–10)	8.4 (2.1)	8.2 (2.1)	8.6 (2)	9.1 (1.4)
Functional status 2 weeks before hospitalization: MBI (0–100)	88.4 (19.1)	83.6 (22.3)	91.5 (16)	89.6 (16.0)
Anxiety: HADS (0–21)	14.2 (7.1)	10.5 (7.1)	16.1 (6.6)	6.5 (4.7)
Depression: HADS (0–21)	17.8 (8.1)	13.7 (7.6)	18.7 (7.1)	9.2 (3.4)
Economic status: lower than average N(%)	167 (79.9%)	111 (87.4%)	50 (98%)	340 (84.8%)
Length of stay in hospital (days)	6.3 (5)	7.8 (11.2)	6 (4.1)	6.2 (3.7)
Severity of illness (NEWS) (0–20)	1.5 (1.7)	1.7 (1.8)	1.5 (1.7)	1.6 (1.7)
Comorbidity score: Charlson (0–22)	2.4 (2.1)	2.4 (1.8)	2.2 (2)	2.2 (1.9)

#### Preliminary analysis

All background characteristics (age, gender, family status, and number of children, as well as functional, cognitive, psychological, and physical health status) were significantly related to at least one of the study variables and thus were included as control variables.

#### Ethno-cultural sample characteristics

About half the participants were “Jews” (54.0%, *N* = 209), and about a third were “Arabs” (32.8%, *N* = 127). Only 13.2% (*N* = 51) were “FSU immigrants.”

#### Informal caregiving during hospitalization

##### Hours of day visits

Almost all participants (98.4%, *N* = 381) had at least one visit from family members or friends during the first 3 days of their hospital stay. The average length of the day visit was 6.6 h (*SD* = 3.6).

##### Night stay

Only 16.0% of participants (*N* = 62) were accompanied by family caregivers during the night (a “stay” was defined as at least 4 h).

##### Type of support

More than half the participants (58.7%, *N* = 227) received instrumental support from their family caregivers during hospitalization, and a similar share (61.2%, *N* = 237) received supervision of instrumental support from hospital staff. Psychological support was most prevalent: 97.4% of hospitalized elders (*N* = 377) reported this kind of support from family and friends during hospitalization. The majority (91.5%, *N* = 354) reported that their family caregivers ensured/supervised medical care.

#### Ethno-cultural differences in informal caregiving

##### Hours of day visits

MANCOVA analyses controlling for all covariates revealed significant differences between ethno-cultural groups (*F* (2, 466) = 4.03, *p* = .019). Bonferroni post-hoc analysis showed that “FSU immigrants” had the shortest visits (estimated average 5.2 h per day) compared with “Jews” (6.6 h per day, *p* = .038) and “Arabs” (7.2 h per day, *p* = .015) (Table 4).

**Table 4:** Study 2: Ethno-cultural differences in informal caregiving (*N* = 387)

	“Jews” (*N* = 209)	“Arabs” (*N* = 127)	“FSU immigrants” (*N* = 51)
	*M* (*SD*)^g^	*M* (*SD*)^g^	*M* (*SD*)^g^
Day visit (hours)	6.6 (0.2)^a^	7.2 (0.4)^b^	5.2 (0.5)^a,b^
Instrumental support (1–5)	2.1 (0.1)	2.1 (0.1)	1.9 (0.2)
Supervision instrumental support (1–5)	2.0 (0.1)	2.1 (0.1)	1.6 (0.2)
Ensuring/supervision medical care (1–5)	3.5 (0.1)^c^	3.7 (0.1) ^d^	2.7 (0.2) ^c,d^
Psychological support (1–5)	3.8 (0.1)^e^	3.8 (0.1)^f^	3.0 (0.2)^e,f^

^a^*p* < 0.05

^b^*p* < 0.05

^c, d, e, f^*p* < 0.001

^g^Adjusted for covariates: age, gender, family status, number of children; cognitive, functional, psychological (anxiety and depression), and economic status; length of stay; severity of illness, comorbidity; participating in intervention

##### Night stay

No statistically significant associations were found between ethno-cultural groups and the possibility of family caregivers’ night stay.

##### *Differences in types of support*

MANCOVA analyses, including all relevant covariates, showed that the category “FSU immigrants” was significantly associated with receiving less ensuring/supervision of medical treatment (*M* = 2.7, *SD* = 0.2) than “Jews” (*M* = 3.5, *SD* = 0.1, *p* < 0.001) or “Arabs” (*M* = 3.7, *SD* = 0.1, *p* < 0.001). “FSU immigrants” were also significantly less likely to receive psychological support from their family caregivers (*M* = 3.0, *SD* = 0.2) than were “Jews” (*M* = 3.8, *SD* = 0.1, *p* < 0.001) or “Arabs” (*M* = 3.8, *SD* = 0.1, *p* < 0.001) (Table 4).

## Discussion

Our study investigated differences in the amount and kind of informal support provided to older Israeli adults during hospitalization, comparing three cultural/ethnic groups that we labeled “Jews” (i.e., Israeli-born Jews or veteran immigrants), “Arabs,” and “FSU immigrants.” Our analysis of unrelated cohorts in different medical centers reveals similar results for patterns of caregiving in these three groups. Results of these studies suggest that the group that differs the most in providing informal support during hospitalization of older adults is “FSU immigrants.”

Informal “FSU immigrants” caregivers stayed fewer hours during the day and were less present during the night in Study 1 than were caregivers for either “Jews” or “Arabs.” A previous study similarly found that FSU immigrants were less often accompanied by family caregivers in the hospital [32]. Our study extended this finding by showing that they were less accompanied during the night and had shorter visits from family caregivers during the day. This can be explained in a number of ways. Jewish Soviet families have traditionally been characterized as having strong family ties and providing functional support [44]. It is possible, then, that FSU family caregivers perceive the hospital visit time as functional. Thus, although they may bring necessary equipment or a favorite food, they do not spend extra time in the hospital [45]. It is also possible that FSU immigrants and their family caregivers hold different views of the health care system. They might be accustomed to the paternalist characteristics of Soviet society and its medical system [46]. In the Soviet system, hospital staff take full responsibility for a patient’s condition, and family caregivers are forbidden to visit except during certain daytime hours*.* Even if family caregivers of elderly patients emigrated in childhood or were born in Israel, they might share the norms of their country of origin [47].

In Study 2, “FSU immigrants” also provided less ensuring/supervision of medical care than the two other groups. This could reflect difficulties communicating with hospital staff; FSU immigrants and their family caregivers may be less fluent in Hebrew. Family caregivers (especially the older generation) may also feel uncomfortable discussing a patient’s condition with medical staff. Consequently, medical staff may discuss health situation and treatment less with FSU family members.

Study 1 demonstrated that family caregivers of “Arab” older adults were more likely to stay during the night than those in the two other ethno-cultural groups. This could be related to the Arabic cultural tradition of respect for older adults. Despite modernization and ongoing changes, adult children in Arab-Israeli society still show relatively high levels of filial piety, a cultural norm that encourages care of parental well-being [48]. Another possible explanation is the extension of family support networks in Arabic families to include larger families with more children, friends, and neighbors. Members of these extended networks often help care for older adults [28], a tendency likely to appear in the hospital setting.

There were no differences between ethno-cultural groups for instrumental and supervision of instrumental support in either study. It seems that the involvement of family caregivers for such support is determined more by the functional and health condition of the patient than by cultural norms and patterns.

Interestingly, the studies had opposite findings for psychological support. The results of Study 2 are in line with other work finding that immigrants from the FSU receive less help. However, in Study 1, immigrants from the FSU reported receiving more psychological support than did Israeli-born and veteran immigrant Jews. It is possible that psychological support was sensitive to the contexts of the different hospitals included in the present work. Future studies could include qualitative data and investigate the meaning of psychological support in different cultures and hospitals.

The main limitation of this research was that information on informal caregiving during hospitalization was self-reported by patients. Future work should take a dyadic perspective and include the viewpoint of family caregivers as well. Moreover, it would be valuable to evaluate family caregivers’ characteristics such as health, level of health literacy, family employment status, and distance from the hospital.

## Conclusion and implications

This study has important practical implications. FSU immigrants seem to receive less support during hospitalization than members of other ethno-cultural groups; this may fall short of the implicit expectations of Israeli health and social care workers [45]. Health care professionals should be aware of the cultural norms affecting these behaviors. This awareness, in turn, could help build mutual understanding, ensure cooperation between family and formal caregivers of older adults, and lead to clearer role distribution and better care.

FSU immigrants often receive language-discordant medical care [48], and their family caregivers should ideally act as a bridge between the patient and the medical system to increase patients’ health literacy and improve hospitalization outcomes [49–51]. However, our results suggest that their family caregivers are less able to fulfill this role and may experience difficulties because of language and cultural gaps; thus, it may be beneficial to overcome these difficulties by using translators in the hospital setting.

Our results emphasize the need for greater awareness among health care workers of the characteristics of ethno-cultural groups, so that they can provide cultureappropriate care and pay attention to cultural issues when communicating with family caregivers.

## Data Availability

The datasets analysed during the current study available from the corresponding author on reasonable request.
